# Mechanistic roles of long non-coding RNAs in gastric cancer therapy resistance

**DOI:** 10.1016/j.ncrna.2025.12.004

**Published:** 2026-01-06

**Authors:** Jiayi Chen, Juanmei Gao

**Affiliations:** a Ningbo Clinical Pathology Diagnosis Center, Ningbo, China; b Department of Obstetrics and Gynecology, Affiliated Hangzhou First People's Hospital, Westlake University School of Medicine, Hangzhou, Zhejiang, China

**Keywords:** Long non-coding RNAs, Gastric cancer, Chemotherapy resistance, Immunotherapy, Targeted therapy

## Abstract

Gastric cancer (GC) remains a leading cause of cancer-related mortality worldwide. While gastrointestinal tumor screening has reduced incidence and mortality, its treatment faces is hindered by challenges including chemotherapy resistance and poor prognosis. Long non-coding RNAs (lncRNAs), a class of non-coding RNAs exceeding 200 nucleotides in length, serve as pivotal regulators in GC pathogenesis and therapeutic resistance. This review comprehensively summarizes the mechanistic roles of lncRNAs in chemotherapy, immunotherapy, and targeted therapy resistance for GC. In chemotherapy, lncRNAs modulate drug sensitivity to fluoropyrimidines (5-FU), platinum-based agents and other chemotherapeutics by regulating autophagy, apoptosis, metabolic reprogramming and DNA damage repair mechanisms, such as HNF1A-AS1, LINC00942 and CRNDE. In immunotherapy, lncRNAs influence immune checkpoint inhibitor efficacy by regulating PD-L1 expression, tumor microenvironment (TME), and macrophage polarization (e.g., LINC01094, MIR4435-2HG). Notably, specific lncRNAs (e.g., LINC00665, HOTAIR) contribute to resistance against HER2-targeted and anti-angiogenic therapies. Although current research remains exploratory, lncRNAs show significant promise as predictive biomarkers and therapeutic targets. Future personalized strategies intergrating lncRNA profiles could help overcome drug resistance and improve patient outcomes.

## 1 Introduction

In recent years, the widespread implementation of upper gastrointestinal cancer screening programs in China has significantly reduced age standardized incidence and mortality rates of esophageal, gastric, and liver cancers [1]. Nevertheless, epidemiological data indicate gastrointestinal malignancies, including gastric cancer (GC), liver cancer, and colorectal cancer, remain the leading causes of cancer-related deaths, second only to lung cancer. Currently, surgical resection is the primary treatment for early-stage GC, with a 5-year survival rate exceeding 90 % [2]. For advanced GC, therapeutic strategies have evolved from conventional chemotherapy to individualized approaches incorporating molecular typing, including targeted therapy combined with chemotherapy or immunotherapy (NCCN Guidelines Version 2.2025 Gastric Cancer). However, chemotherapy remains the mainstay for patients lacking actionable therapeutic targets. Despite demonstrated efficacy, drug resistance significantly limits clinical benefits. Chemoresistance mechanisms in GC involve complex molecular pathways including autophagy activation, ferroptosis suppression, apoptotic pathway dysregulation, cell cycle checkpoint disruption, aberrant proliferation signaling, and drug target gene mutations [3]. Thus, in-depth investigation of resistance mechanisms is crucial for developing novel therapeutic strategies and improving patient outcomes.

Long non-coding RNAs (lncRNAs), a class of RNA exceeding 200 nucleotides in length without protein-coding capacity [4]. The discovery of lncRNAs represents a paradigm shift in genomics. Initially dismissed as transcriptional noise, their functional importance became evident with the advent of high-throughput sequencing technologies and landmark projects such as Encyclopedia of DNA Elements (ENCODE), which demonstrated pervasive transcription across the human genome and revealed that approximately 80 % of it possesses biochemical activity, including lncRNAs [5]. Further annotation of lncRNAs has demonstrated that they are generated through mechanisms analogous to those of protein coding genes, exhibiting canonical gene structures and functions associated with histone modifications [6]. This discovery has significantly broadened the understanding of lncRNAs. Consequently, a growing number of lncRNAs have been identified, the number of human lncRNAs has increased to 173112 [7]. Researchers have systematically classified lncRNAs based on their features, such as transcript length, functions, and protein coding RNA resemblance [8]. The vast and diverse repertoire of lncRNAs precisely regulates gene expression at multiple levels, including epigenetic, transcriptional, and post-transcriptional regulation, governing key biological processes such as cell differentiation, development, and disease pathogenesis [9]. Most lncRNAs act as oncogenes or tumor suppressors, regulating cancer initiation and progression either by directly modulating the activity, stability, or interactions of key signaling pathway proteins, or by functioning as molecular sponges for microRNAs (miRNAs) and mRNAs [[10], [11], [12], [13], [14]]. These molecules precisely modulate cell proliferation, differentiation, metastasis, and therapy resistance via endogenous competing RNA (ceRNA) networks, protein activity modulation, and chromatin remodeling [[15], [16], [17], [18], [19]]. Aberrant lncRNA expression is closely associated with GC development and directly influences chemosensitivity, radiotherapy response, and targeted therapy efficacy [3,18,20]. Despite significant advances, most studies remain confined to basic research and requiring clinical validation. This review systematically summarizes mechanistic insights into the roles of lncRNAs in GC chemotherapy, immunotherapy, and targeted therapy resistance, evaluating their potential as novel biomarkers and therapeutic targets to advance precision medicine.

## 2 Molecular regulatory mechanisms of non-coding RNA (ncRNA)

### 2.1 Molecular regulatory mechanisms of lncRNAs

**Fig. 1:**
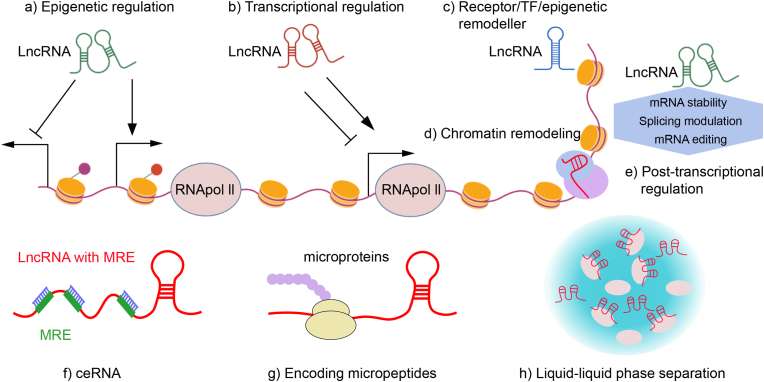
Molecular Regulatory Mechanisms of LncRNAs. This figure systematically illustrates the multilevel gene regulatory network mediated by lncRNAs through diverse molecular interaction modes, covering classical regulatory pathways and novel regulatory mechanisms, as detailed below: epigenetic regulation (a), transcriptional regulation (b, c, d), post-transcriptional regulation (e, f), and novel regulatory mechanisms (g, h).

The core functional mechanisms of lncRNA lie in the three major interaction modes of RNA–DNA, RNA–RNA, and RNA–protein, these interactions form a hierarchical gene regulatory network encompassing epigenetic modification, transcriptional, and post-transcriptional modifications, laying the foundation for the extensive involvement of lncRNAs in physiological and pathological processes [21,22]. Notably, compared with traditional classification methods, genomic localization based on classification (e.g., enhancer RNAs (eRNAs), antisense lncRNAs) is more suited to matching their functional specificity, meanwhile, the encoding of functional micropeptides and regulation mediated by liquid-liquid phase separation (LLPS) have emerged as key research directions in recent years [21]. In addition, lncRNA functions exhibit significant spatiotemporal specificity, playing multiple roles in developmental programming, stress response, and disease progression. Abnormal activation or silencing of lncRNA regulatory networks is an important inducing factor for pathological processes such as cancers and neurodegenerative diseases [22]. These mechanisms complement each other and collectively constitute the multilevel and multidimensional regulatory network of lncRNAs (Fig. 1). This enables its extensive participation in genomic spatial organization, cellular structural homeostasis, and diverse physiological and pathological processes, establishing lncRNA as an indispensable core molecule within the intracellular gene regulatory network. It also lays a crucial theoretical foundation for exploring the clinical translational value of lncRNA regulatory mechanisms.

At the level of epigenetic regulation, the molecular scaffolding function of lncRNAs is one of the core mechanisms mediating long term gene silencing or activation [23]. As molecular scaffolds, lncRNAs recruit epigenetic modification complexes such as Polycomb Repressive Complex 2 (PRC2) and lysine-specific demethylase 1 (LSD1) to mediate histone modifications and regulate gene transcription [24]. They also participate in DNA methylation regulation by targeting the localization of DNA methyltransferases/Ten-Eleven translocation proteins (DNMTs/TETs) interfering with DNMT cofactor metabolism, or regulating the expression of DNMTs/TETs, thereby playing a central role in development and tumorigenesis [25]. Additionally, lncRNAs interact with the SWI/SNF complex through direct binding or recruitment to regulate chromatin remodeling and cancer progression [26]. In transcriptional regulation, activating lncRNAs including eRNAs can recruit regulatory factors such as the Mediator complex to stabilize the enhancer-promoter chromatin loop structure, thereby facilitating transcription complex assembly and transcriptional activation of target genes [27]. Simultaneously, lncRNAs can remodel the transcriptional programs of key genes through competitive inhibition or synergistic activation of transcription factors, or alternatively directly bind to RNA polymerase II (Pol II) to prevent its binding to target gene promoters or inhibit its transcriptional elongation activity, thereby repressing gene transcription [23]. At the post-transcriptional regulatory level, the ceRNA mechanism represents the most classic regulatory mode of lncRNAs, whereby lncRNAs competitively bind to miRNAs via sharing miRNA response elements (MREs) to alleviate miRNA-mediated inhibition of target mRNAs and thereby regulate gene expression [28]. Moreover, lncRNAs can directly modulate alternative splicing, stability, and translation efficiency of target mRNAs, forming an elaborate regulatory network during the post-transcriptional phase of gene expression [22]. Recent studies have further revealed two novel lncRNA mediated gene regulatory mechanisms. First, certain lncRNAs encode functional micropeptides via small open reading frames (sORFs), which typically consist of dozens to more than a hundred amino acids. These micropeptides interact with proteins to participate in RNA splicing, stability regulation, cellular metabolism, and signal pathway modulation, thereby affecting key biological processes associated with cancer development [29]. Second, lncRNAs mediate LLPS-driven regulation by leveraging their structural features characterized by abundant multivalent repeat sequences and intrinsic phase separation potential, interacting with other RNA and protein molecules to assemble functional nuclear bodies that precisely regulate biological processes including gene transcription, RNA processing, and genomic stability maintenance [21].

### 2.2 Circular RNAs (circRNAs): complementary mechanisms and synergistic regulation with lncRNAs

As core members of the ncRNA family, circRNAs and lncRNAs share some regulatory modes. Both participate in gene regulation through the ceRNA mechanism, protein interactions, and functional micropeptide encoding, serving as key synergistic factors in gene regulatory networks [30,31]. However, the unique covalently closed circular structure of circRNA distinguishes it from linear lncRNAs and confers core functional specificity. This structure confers high resistance to degradation by RNA exonucleases, resulting in significantly greater intracellular stability compared to linear RNAs, this enables its persistent accumulation and longterm regulatory function, laying a structural foundation for its potential as a stable biomarker [32,33]. Structure determines function, the circular conformation of circRNAs gives them two unique advantages. First, high efficiency as molecular scaffolds. Compared with the binding domains formed by the dynamic folding of linear RNAs, the circular backbone of circRNAs can provide a more optimized spatial arrangement and synergistic binding interface for multiple interacting proteins, facilitating the precise assembly and regulation of functional ribonucleoprotein complexes [34]. Second, unique translational potential. Some circRNAs carrying internal ribosome entry site (IRES) elements can initiate cap-independent translation under specific physiological stress or pathological signal stimulation, generating functional small proteins that participate in key biological processes such as feedback regulation of cell signaling pathways and remodeling of energy metabolism [35]. This is in sharp contrast to the functional pathways of most lncRNAs.

It can be seen that circRNAs are centered on a closed-loop structure, deriving a regulatory mode with distinctive characteristics in stability, scaffolding ability, and translation mechanism, and form functional complementarity with lncRNAs. The ncRNA synergistic regulatory network formed by the two not only reshapes our understanding of the concept of noncoding, but also reveals the complexity and precision of noncoding genome regulation. From the core regulatory role of lncRNAs to the complementary function of circRNAs, the diverse regulatory modes mediated by ncRNAs are essentially evolutionary adaptations of the organism in response to physiological and pathological demands. This also provides a more comprehensive theoretical foundation for further dissecting the molecular mechanisms of diseases such as gastric cancer and for developing novel diagnostic biomarkers and therapeutic targets.

## 3 LncRNAs and chemotherapy

**Fig. 2:**
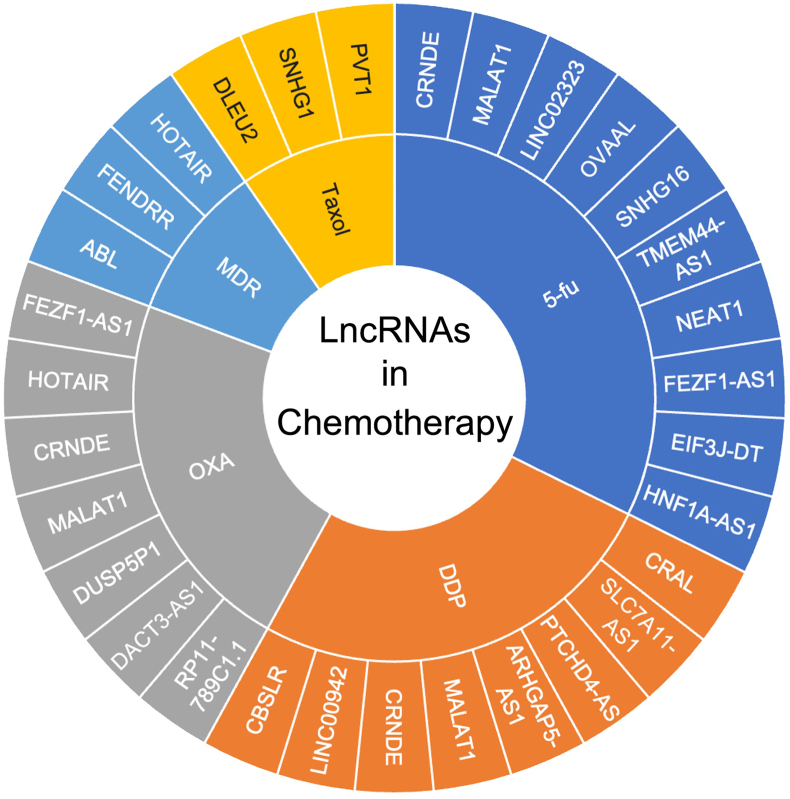
Lncrnas related to chemotherapy drugs in gastric cancer.

Chemotherapy is the primary treatment modality for unresectable or advanced postoperative GC, with common agents including fluoropyrimidines (5-FU), platinum and paclitaxel (Taxol). Despite improved survival and quality of life, drug resistance poses significant clinical challenges. LncRNAs are pivotal regulators of tumor drug resistance. This section mechanistically delineates lncRNA involvement in GC chemotherapy resistance, as summarized in Fig. 2 and detailed in Table 1.

**Table 1:** The LncRNAs in chemotherapy.

LncRNAs	Expression	Mechanisms	Drug	Reference
HNF1A-AS1	Up	Regulating miR-30b-5p/EIF5A2 axis; promote EMT	5-Fu	[36]
EIF3J-DT	Up	Regulating ATG14, promote autophagy	5-Fu	[37]
FEZF1-AS1	Up	Regulating ATG5, promote autophagy	5-Fu/OXA	[38]
NEAT1	Up	Interacts with hnRNPA2B1, maintain stemness property via Wnt/β‐catenin pathway	5-Fu	[39]
TMEM44-AS1	Up	Inhibiting the p53 pathway	5-Fu	[40]
SNHG16	Up	Regulating miR-506-3p/PTBP1 axis; promote glycolysis metabolism	5-Fu	[41]
OVAAL	Up	Enhance pyrimidine biosynthesis; promote proliferation	5-Fu	[42]
LINC02323	Up	Regulating miR-139-3p	5-Fu	[43]
CBSLR	Up	Regulating CBSLR/YTHDF2/CBS axis; inhibite ferroptosis	DDP	[45]
LINC00942	Up	Regulating LINC00942/MSI2/c-Myc axis; suppressing MSI2 degradation	DDP	[18]
HOTAIR	Up	Regulating HOTAIR/miR-195-5p/ABCG2 axis; promote proliferation; Regulating HOTAIR/miR-17‐5p/PTEN axis;	OXA MDR	[46] [56]
CRNDE	Up	Transfer of TAM‐derived exosomes Regulating autophagy	DDP OXA/5-Fu	[47] [19,59]
MALAT1	Up	Promote glycolysis; promote autophagy	OXA 5-Fu/DDP	[48] [49,50]
ARHGAP5-AS1	Up	Impaired autophagy	DDP	[51]
DUSP5P1	Up	Activating focal adhesion and MAPK pathways	OXA	[52]
PVT1	Up	Regulatingcell cycle and apoptosis	Taxol	[53]
SNHG1	Up	Regulating glucose metabolic	Taxol	[54]
DLEU2	Up	Regulating glucose metabolic	Taxol	[55]
FENDRR	Up	Regulating FENDRR/miR-4700-3p/FOXC2 axis	MDR	[57]
ABL	Up	Blocking apoptosome assembly	MDR	[58]
PTCHD4-AS	Down	Induced DNA damage	DDP	[60]
DACT3-AS1	Down	Promotes ferroptosis	OXA	[61]
SLC7A11-AS1	Down	Regulating GSH	DDP	[62]
RP11-789C1.1	Down	Regulating ATR/CHK1 pathway	OXA	[63]
CRAL	Up	Regulating miR-505/CYLD/AKT axis	DDP	[64]

### 3.1 LncRNAs and 5-FU chemoresistance

5-FU is a cornerstone agent for perioperative and palliative chemotherapy in GC, with complex and highly heterogeneous resistance mechanisms. In 5-FU resistance, multiple lncRNAs exhibit upregulated expression in resistant GC models, such as HNF1A-AS1, EIF3J-DT, NEAT1, and TMEM44-AS1. Specifically, HNF1A-AS1 promotes chemoresistance by activating epithelial-mesenchymal transition (EMT), while its silencing restores drug sensitivity [36]. Both EIF3J-DT and FEZF1-AS1 induce chemotherapy resistance through autophagy enhancement [37,38]. NEAT1 stabilizes via m6A-dependent interaction with hnRNPA2B1, subsequently activating the Wnt/β-catenin pathway and exacerbating 5-FU resistance [39]. TMEM44-AS1 confers resistance by inhibiting the p53 signaling pathway to block apoptosis, and its downregulation can reverse 5-FU resistance [40]. Furthermore, studies reveal that SNHG16 promotes glycolysis metabolism by forming a SNHG16/miR-506-3p/PTBP1 ceRNA network, leading to enhanced drug resistance, while SNHG16 silencing significantly improves 5-FU sensitivity [41]. Bioinformatics analyses demonstrate that OVAAL aggravates resistance by enhancing pyrimidine biosynthesis and correlates with poor patient prognosis [42]. Clinical cohort data confirm that LINC02323 overexpression associates with chemoresistance and malignant progression, its depletion enhances 5-FU efficacy by inhibiting miR-139-3p mediated apoptosis suppression [43].

### 3.2 LncRNAs and platinum resistance

Platinum agents, either as monotherapy or in combination with other chemotherapeutic drugs, represent the standard first-line treatment for advanced GC, combination regimens enhance therapeutic efficacy [44]. Consequently, research on platinum drugs (particularly cisplatin [DDP] and oxaliplatin [OXA]) is more extensive compared to other chemotherapeutic agents. Nevertheless, platinum resistance remains a major cause of clinical treatment failure. Recent studies have revealed that lncRNAs regulate platinum resistance through diverse molecular mechanisms, offering new perspectives for overcoming chemoresistance. CBSLR forms a CBSLR/YTHDF2/CBS axis in hypoxic microenvironments, reducing cystathionine β-synthase (CBS) mRNA stability and promoting ACSL4 degradation to drive DDP resistance [45]. LINC00942 sustains chemoresistance through a MSI2/c-Myc axis that maintains cancer stemness and inhibits apoptosis [18]. The well characterized HOTAIR sponges miR-195-5p to derepress ABCG2, enhancing OXA resistance [46].

In addition to the ceRNA mechanism, exosome-mediated lncRNA transfer plays a pivotal role in platinum resistance. Studies reveal that M2-type tumor-associated macrophages (M2 TAMs) in the tumor microenvironment can deliver CRNDE to GC cells via exosomes, thereby reducing DDP sensitivity through PTEN expression suppression [47]. Similarly, MALAT1 is transmitted also intercellularly through exosomes, upon entering GC cells, it enhances tumor growth and metastatic potential by promoting aerobic glycolysis and significantly increasing OXA resistance [48]. Notably, MALAT1 drives multidrug resistance via autophagy activation [49,50]. Epigenetically, ARHGAP5-AS1 upregulation in resistant cells activates ARHGAP5 transcription to promote DDP resistance [51], and DUSP5P1 stimulates focal adhesion/MAPK pathways to enhance OXA resistance and metastasis [52]. These findings provide novel insights into lncRNA mediated chemoresistance through epigenetic regulation.

### 3.3 LncRNAs and resistance to other chemotherapeutic agents

Beyond 5-FU and platinum agents, lncRNAs have been implicated in resistance to various other chemotherapeutic agents used in GC treatment. Research demonstrates that lncRNA PVT1 is highly expressed in GC cells and strongly correlates with Taxol resistance [53]. Silencing PVT1 expression significantly enhances Taxol sensitivity, manifested by increased apoptosis and suppressed proliferative/migratory capacities. Similarly, SNHG1 [54] and DLEU2 [55] modulate Taxol responsiveness through glucose metabolic pathway regulation. It is notable that HOTAIR is not only involved in oxaliplatin resistance [46], but also confers cross-resistance to multiple chemotherapeutics (DDP, doxorubicin[ADR], mitomycin, and 5-FU) by simultaneously inhibiting apoptosis and promoting proliferation [56]. In multidrug resistance studies, FENDRR exhibited consistent upregulation and mechanism studies have shown FENDRR acts as a molecular sponge for miR-4700-3p to upregulate the proto-oncogene FOXC2 and driving chemoresistance [57]. Furthermore, lncRNA ABL promotes GC cell proliferation and drug resistance by competitively disrupting APAF1 cytochrome *c* interaction. This blockade impairs apoptosome assembly and subsequent Caspase-9/3 activation, ultimately inhibiting the intrinsic apoptosis pathway [58].

### 3.4 LncRNAs and chemosensitivity

In contrast to chemotherapy resistance promoting lncRNAs, research on lncRNAs that enhance chemosensitivity in GC remains limited, though several key regulators have recently been identified. In OXA and 5-FU resistant GC cells, CRNDE suppression by E2F6 enhances autophagy and resistance to OXA and 5-FU, but restoring CRNDE or inhibiting SRSF6 reverses this effect [19,59]. DDP induced DNA damage transcriptionally upregulates PTCHD4-AS (normally downregulated in GC), which then binds MSH2-MSH6 to trigger the ATM/p53/p21 pathway, ultimately suppressing proliferation and sensitizing cells to DDP [60]. DACT3-AS1 is also under expressed in GC tissues and associates with poor prognosis, mechanistic studies reveal that cancer associated fibroblast (CAF) derived exosomes transfer DACT3-AS1 to suppress miR-181a-5p/SIRT1, inducing ferroptosis and OXA sensitization [61]. SLC7A11-AS1 deficiency in GC cells upregulates xCT to promote glutathione (GSH) synthesis, scavenging drug induced reactive oxygen species (ROS) and conferring DDP resistance. Conversely, SLC7A11-AS1 restoration blocks xCT through miR-33a-5p regulation, rebalancing oxidative stress to suppress tumor growth and reverse chemoresistance [62]. In addition, RP11-789C1.1 is downregulated in both GC tissues and cell lines, functional studies demonstrate that RP11-789C1.1 overexpression inhibits the ATR/CHK1 pathway, inducing DNA damage and apoptosis to enhance OXA efficacy [63]. Similarly, cisplatin resistance-associated lncRNA (CRAL) enhances DNA damage by sponging miR-505 to upregulate CYLD and inhibit AKT [64]. These findings position lncRNAs as promising targets for overcoming chemoresistance.

There is no doubt that lncRNAs play a pivotal role in chemotherapy resistance in GC. Dysregulated lncRNAs mediate key mechanisms of chemoresistance by influencing DNA repair, apoptosis, and signaling pathways, establishing themselves as critical molecular determinants in this process. Although current research has revealed their significant functions, several challenges remain. For instance, a single lncRNA may contribute to drug resistance through multiple mechanisms, or a specific resistance mechanism may be co-regulated by hundreds of lncRNAs. This complexity hinders the clinical translation of lncRNA targeted therapies. Furthermore, achieving efficient, specific, and stable delivery of lncRNA targeting drugs to GC cells in vivo remains a major hurdle. Future success will depend on our ability to overcome technical limitations, identify the most critical nodes within these complex networks, and conduct in-depth investigations into the role of lncRNAs in chemotherapy resistance within the specific molecular context of GC.

## 4 LncRNAs and immunotherapy

Immunotherapy, particularly immune checkpoint inhibitors (ICIs), has transformed GC management. However, therapeutic efficacy remains limited by tumor heterogeneity and the immunosuppressive tumor microenvironment (TME) [65]. The TME serves as a critical regulatory network in tumorigenesis and progression, comprising diverse cellular components including tumor cells, immune cells, CAFs, and others, these elements interact through intricate mechanisms to drive tumor initiation, progression, angiogenesis, and metastasis [66]. Current TME targeting strategies focus on macrophage reprogramming, extracellular matrix (ECM) remodeling, extracellular vesicle (EV) modulation, cell therapies and ICIs [67]. Notably, ICIs targeting key immunosuppressive pathways (particularly PD-1/PD-L1 and CTLA-4 axes), such as the PD-1 inhibitor pembrolizumab and nivolumab, have demonstrated remarkable clinical efficacy across multiple malignancies [68].

**Fig. 3:**
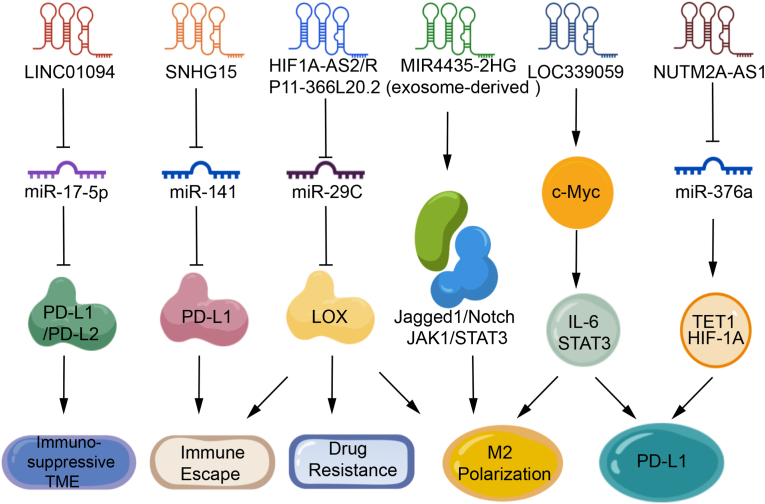
The mechanism of LncRNAs in immunotherapy for gastric cancer.

LncRNAs critically regulate ICI response and TME dynamics. Multiple algorithm-driven analyses of TCGA data have revealed significant correlations between specific lncRNA signatures and immunotherapy responses (Fig. 3). For example, an m6A-related lncRNA model associates low-risk patients with enhanced CD4^+^ Th1 infiltration and elevated PD-1/LAG3, correlating with ICI sensitivity [69]. Similarly, the DNA damage repair associated lncRNA risk model revealed that low-risk patients exhibited higher tumor mutational burden (TMB) and increased cytotoxic T lymphocyte (CTL) activity, resulting in improved clinical outcomes following anti-PD-L1 treatment [70]. Particularly, TME analysis using the ESTIMATE algorithm identified that the low stromal score subtype showed greater responsiveness to ICIs, whereas in high stromal score subtypes, lncRNA BPIFB2 potentially mediated resistance to PD-1/PD-L1 blockade by inducing EMT [71]. Furthermore, multiple lncRNA subtypes including ferroptosis related, immune phenotype score related, and EV related have been consistently associated with immunotherapy response [[72], [73], [74], [75], [76]]. However, their precise mechanistic underpinnings require further experimental validation.

Accumulating experimental evidence has demonstrated that lncRNAs modulate immune checkpoint molecules through ceRNA networks. Specifically, LINC01094 upregulates PD-L1 and PD-L2 by sponging miR-17-5p to establish an immunosuppressive TME, and TCGA data analysis suggests its potential association with CD8^+^ T cell dysfunction and M2 macrophage polarization [77]. SNHG15 upregulates PD-L1 expression by inhibiting miR-141 and promotes immune escape of GC cells [78]. NUTM2A-AS1 was found to regulate PD-L1 via the miR-376a/TET1/HIF-1A axis, and its overexpression enhances DDP resistance in GC cells, suggesting potential synergistic effects between chemotherapy and immunotherapy [79]. Although current research on lncRNAs in GC immunotherapy remains exploratory, accumulating evidence underscores their crucial value in predicting therapeutic response, modulating the immune microenvironment, and overcoming drug resistance.

Tumor associated macrophages (TAMs), as a key component of the tumor microenvironment, play multifaceted pro-cancer roles in tumor progression, including promoting cancer cell proliferation, angiogenesis, and metastasis [80,81]. Studies have shown that tumor cells can recruit macrophages and induce their polarization toward the M2 phenotype through the secretion of inflammatory factors, thereby shaping an immunosuppressive microenvironment, blocking M2 polarization and significantly inhibits tumor growth [82]. Therefore, targeted modulation of TAM polarization has become an important research direction in tumor immunotherapy. Recent investigations have uncovered the critical role of lncRNAs in regulating TAM polarization and GC immune escape. For instance, exosome-derived lncRNA MIR4435-2HG promotes M2 macrophage polarization by activating the Jagged1/Notch and JAK1/STAT3 signaling pathways, thereby accelerating GC progression [83]. Additionally, the HIF1A-AS2/miR-29c/LOX regulatory axis has been shown to promote M2 polarization, enhance immune escape and chemoresistance, and lead to a poor prognosis in GC patients [84]. Another retrospective study showed that LOC339059 was downregulated in GC tissues. Its deficiency activates the c-Myc/IL-6/STAT3 signaling pathway, leading to PD-L1 upregulation and enhanced M2 macrophage polarization, while restoring LOC339059 expression could inhibit this process and exert a tumor suppressor effect [85]. Beyond these findings, additional lncRNAs including ElNF1-AS1 [86], LINC00665 [87], MIR181A2HG [88], also modulate macrophage function through distinct molecular mechanisms and promote the progression of GC.

Based on current evidence, lncRNAs act as pivotal yet complex nodal regulators in the molecular network underlying immunotherapy resistance in GC. They drive resistance through mechanisms such as modulating immune checkpoint expression and shaping an immunosuppressive TME. However, research in this field remains limited by simplistic models and superficial mechanistic insights. Existing models fail to fully recapitulate the complexity of the human immune system, and the high cell and tissue specificity of lncRNAs presents challenges for developing broad-spectrum targeting strategies. Future studies should leverage multi-omics integration and more sophisticated models to investigate lncRNA functions in immune regulation within physiologically relevant contexts, such as immune cell tumor organoid co-cultures or humanized patient derived xenograft (PDX) models. Additionally, combining lncRNA targeting approaches with ICIs may offer promising strategies to overcome immunotherapy resistance in GC.

## 5 LncRNAs and targeted therapy

Targeted therapy represents a cornerstone of GC precision medicine, though clinical translation faces challenges. Currently, the targeted drugs approved by major global regulatory agencies (FDA, EMA, NMPA) for GC remain primarily focused on two categories: anti-HER2 therapies and anti-angiogenic agents. Of particular note, the latest versions of the NCCN (Version 2.2025 Gastric Cancer) and CSCO (Clinical guidelines for the diagnosis and treatment of gastric cancer, 2025) guidelines have incorporated CLDN18.2 targeted agents into their recommendations, providing a novel therapeutic option for GC treatment.

In anti-HER2 treatment, trastuzumab is used as the basic drug, with its resistance mechanism is becoming increasingly in-depth. Emerging evidence highlights the pivotal role of lncRNAs in mediating drug resistance. LINC00665 promotes tumorigenesis and resistance by sponging miR-199b-5p to upregulate SERPINE1, thereby activating the PI3K/AKT pathway [89]. The well-characterized HOTAIR not only serves as a prognostic biomarker but also forms a ceRNA network with miR-331-3p and HER2 to drive disease progression [90]. Subsequent studies revealed its additional role in competitively binding miR-330 to upregulate ERBB4, activating PI3K/AKT signaling and conferring resistance [91]. In lapatinib resistance models, chronic drug exposure induces p-STAT3 nuclear translocation in HGC-27 cells, upregulating NONHSAT160169.1, which enhances tumor stem cell properties and resistance via the hsa-let-7c-3p/SOX2 axis [92]. At present, except for anti-HER2 drugs, the research on other targeted therapeutic drugs and lncRNAs remains limited. Existing studies have shown that CRART16 drives angiogenesis and bevacizumab resistance through the miR-122-5p/c-Fos/VEGFD axis [93]. These discoveries provide novel molecular insights for overcoming targeted therapy resistance in GC.

CLDN18.2 has emerged as a prominent molecular target in GC targeted therapy, with zolbetuximab being a key focus in both clinical research and translational studies. Large scale clinical trials and translational investigations have consistently demonstrated that zolbetuximab whether administered as monotherapy or in combination confers significant survival benefits in patients with GC [[94], [95], [96], [97], [98]]. The antitumor mechanism of CLDN18.2 targeting agents primarily involves antibody-dependent cellular cytotoxicity (ADCC) and complement-dependent cytotoxicity (CDC) effects [99]. Although no direct studies have yet explored the relationship between lncRNAs and ADCC/CDC mechanisms, insights drawn from relevant literature suggest plausible regulatory roles of lncRNAs. LncRNAs may influence the activity of immune effector cells by modulating inflammatory and immune related signaling pathways such as NF-κB and JAK/STAT [100], thereby affecting ADCC/CDC efficacy. Alternatively, they may regulate the expression level of Claudin18.2 through pathways like PI3K/AKT and ERK/MAPK [100], consequently altering antibody binding efficiency and the cytotoxic outcomes of ADCC/CDC.

Current research predominantly focuses on the role of lncRNAs in fundamental biological processes such as inflammation and apoptosis. Future studies should prioritize the clinical translation of lncRNAs, including the identification of lncRNA biomarkers capable of predicting the efficacy of antibody-based drugs, as well as exploring combination strategies targeting specific lncRNAs (e.g., MALAT1, XIST) with antibody therapies to enhance sensitivity and overcome drug resistance.

## 6 Discussion

**Fig. 4:**
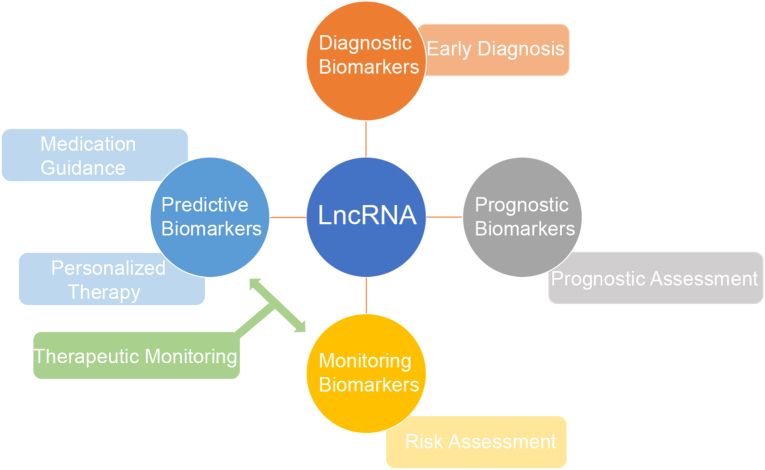
The Future of lncRNA in Precision Cancer Therapy.

Current research on lncRNAs primarily relies on preclinical models and bioinformatics approaches. However, the further integration of lncRNA analysis into clinical applications holds promise for improving enhancing early diagnosis, enabling precision therapy, and improving patient prognosis (Fig. 4). For instance, investigating the characteristics of early GC through big data analytics and utilizing liquid biopsy to detect specific lncRNA signatures in blood [101] or EV-lncRNA profiles [102,103] could effectively identify patients with resectable early stage disease, thereby increasing opportunities for curative surgery and prolonged survival. Therefore, accelerating the identification of clinically relevant lncRNA signatures will significantly enhance the potential for clinical implementation of liquid biopsy technologies.

Furthermore, lncRNA targeted therapies represent a promising future direction. ncRNA based therapeutics have already achieved clinical success, most notably with miRNAs [104]. The lncRNA H19 targeting agent BC-819 (H19-DTA) has completed clinical trials in ovarian [105], bladder [106], and pancreatic cancers [107] with favorable results, though it has not yet received clinical approval. Prior to the development of CRISPR technology, antisense oligonucleotides (ASOs) and small interfering RNAs (siRNAs) were common and effective methods for RNA knockdown. The advent of CRISPR technology, particularly the CRISPR-Cas13 system, has demonstrated superior efficiency and specificity in RNA targeting [108,109], offering robust support for future RNA targeted therapies. However, the complexity of lncRNA regulatory networks suggests that targeting a single lncRNA may be insufficient to achieve durable therapeutic effects. Consequently, exploring the synergistic interactions between lncRNAs and various therapeutic modalities, including chemotherapy, immunotherapy, and targeted therapy, may lead to the development of effective combination regimens that maximize therapeutic efficacy.

**Fig. 5:**
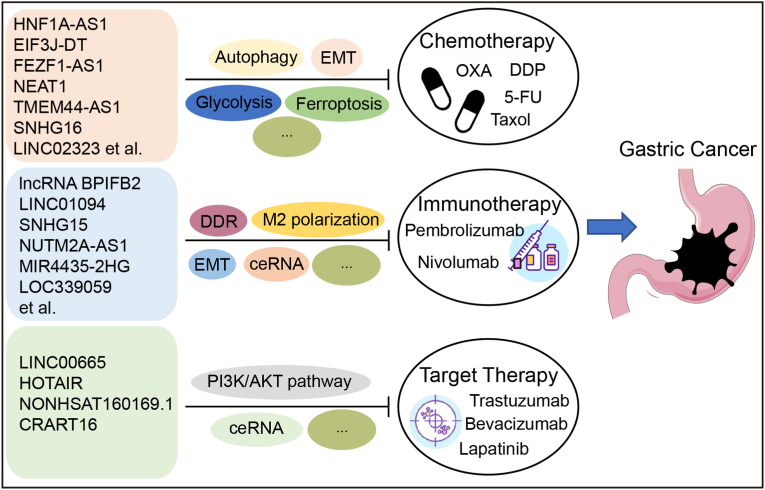
The diagram shows that the resistance mechanisms of LncRNAs chemotherapy, immunotherapy and targeted therapy.

Although the clinical translation of lncRNAs faces significant challenges, research in this area has opened promising new avenues for precision medicine in GC. This review has summarized the roles of lncRNAs in GC therapy resistance, with a focus on their mechanistic roles in chemoresistance, immunotherapy resistance, and targeted therapy evasion, and underscores their dual potential as predictive biomarkers and therapeutic targets (Fig. 5). Mounting evidence demonstrates that lncRNAs play crucial roles in treatment resistance by regulating key processes such as DNA damage repair, apoptosis, and tumor microenvironment remodeling, rendering them compelling candidates for biomarker development and targeted intervention. Future research should prioritize integrating the biological functions of lncRNAs with advanced drug development platforms and bioinformatics approaches to accelerate the development of lncRNA derived biomarkers and the clinical application of targeted therapies, thereby improving early diagnosis, overcoming treatment resistance, and enhancing patient prognosis.

## CRediT authorship contribution statement

**Jiayi Chen:** Writing – original draft, Funding acquisition, Formal analysis, Data curation, Conceptualization. **Juanmei Gao:** Writing – review & editing, Writing – original draft, Formal analysis, Data curation, Conceptualization.

## Funding

This work was supported by the Medical Science and Technology Program in Ningbo (2023Y34), 10.13039/100007834Ningbo Natural Science Foundation (2021J277) and Ningbo Top Medical and 10.13039/100005622Health Research Program (2023010211).

## Declaration of competing interest

The authors declare that they have no known competing financial interests or personal relationships that could have appeared to influence the work reported in this paper.
